# The value of quantitative and a new qualitative color pattern shear wave elastography for the differentiation of ACR TI-RADS 4 or 5 category thyroid nodules measuring ≤10 mm

**DOI:** 10.3389/fendo.2023.1275256

**Published:** 2024-01-08

**Authors:** Ai-jiao Yi, Wei-Wei Yang, Xin-Wu Cui, Christoph F. Dietrich, Bin Wang

**Affiliations:** ^1^ Department of medical ultrasound, Yueyang Central Hospital, Yueyang, China; ^2^ Department of Medical Ultrasound, Tongji Hospital, Tongji Medical College, Huazhong University of Science and Technology, Wuhan, China; ^3^ Department Allgemeine Innere Medizin, Kliniken Hirslanden Beau Site, Salem und Permanence, Bern, Switzerland

**Keywords:** thyroid nodule, ultrasonography, shear wave elastography, ACR TI-RADS, color pattern

## Abstract

**Objective:**

This study aims to evaluate the diagnostic performance of quantitative shear wave elastography (SWE) and a new qualitative color pattern SWE for the differentiation of benign and malignant American College of Radiology Thyroid Imaging, Reporting, and Data System (ACR TI-RADS) 4 or 5 category thyroid nodules measuring ≤10 mm.

**Materials and methods:**

From May 2020 to July 2022, a total of 237 patients with 270 thyroid nodules were enrolled, and conventional ultrasound and SWE examinations were performed for each patient. Each ACR TI-RADS 4 or 5 category thyroid nodule measuring ≤10 mm was evaluated by quantitative SWE and a new qualitative color pattern SWE. The diagnostic performance of quantitative SWE parameters, the new qualitative color pattern SWE, and the combination of SWE with ACR TI-RADS, respectively, for the differentiation of benign and malignant ACR TI-RADS 4 or 5 category thyroid nodules measuring ≤10 mm was evaluated and compared.

**Results:**

Among 270 thyroid nodules in 237 patients, 72 (26.67%) thyroid nodules were benign and 198 (73.33%) thyroid nodules were malignant. The qualitative color pattern SWE showed better diagnostic performance than the quantitative SWE parameters. When combining the qualitative color pattern SWE with ACR TI-RADS scores, with the optimal cutoff value of the total points ≥8, the thyroid nodules were considered malignant. The sensitivity, specificity, accuracy, and AUC were 89.90%, 56.94%, 81.11%, and 0.820 (95% CI: 0.768–0.864), respectively. Compared with using qualitative color pattern SWE alone, the combination of qualitative color pattern SWE and ACR TI-RADS had better diagnostic performance, which was significantly different (*p* < 0.05).

**Conclusion:**

The combination of qualitative SWE color patterns and ACR TI-RADS had high sensitivity and accuracy, which might be a convenient and useful method to differentiate benign and malignant ACR TI-RADS 4 or 5 category thyroid nodules measuring ≤10 mm. It would be helpful for the management of thyroid nodules and improving prognosis.

## 1 Introduction

Thyroid nodules are a common clinical disease with a prevalence of 19%–68% in the population, and 7%–15% of these nodules are thyroid cancers (1). Ultrasound is the first-line modality to screen thyroid nodules. Based on the features of conventional ultrasound including composition, echogenicity, shape, margin, or echogenic foci, the American College of Radiology Thyroid Imaging, Reporting, and Data System (ACR TI-RADS) is widely used for risk stratification and fine-needle aspiration (FNA) recommendation (2). However, there were overlaps in conventional ultrasound features between benign and malignant thyroid nodules, and thyroid nodules measuring ≤10 mm were not routinely recommended for FNA according to the ACR TI-RADS. Thus, it is important to find a non-invasive and useful method to differentiate benign from malignant ACR TI-RADS 4 or 5 thyroid nodules measuring ≤10 mm.

In recent years, shear wave elastography (SWE) has been used to evaluate the stiffness of thyroid nodules (3, 4). Some studies have reported that the elastography index of thyroid cancer is higher than that of benign thyroid nodules (3, 5, 6), which makes SWE a promising method to differentiate benign from malignant thyroid nodules. However, several characteristics of thyroid nodules may affect the results of the quantitative SWE parameters (7–9), including nodule size or calcification. Li et al. (7) found that nodule size affected the optimal *E*_max_ cutoff value of SWE. Chen et al. (8) reported that the mean and maximum shear wave speed (SWS), respectively, of thyroid nodules increases progressively from non-calcification to micro-calcification and macro-calcification groups. Moreover, the optimal quantitative SWE parameters to differentiate benign and malignant thyroid nodules in different studies were varied.

Qualitative color pattern SWE could evaluate the stiffness of the whole thyroid nodule, which might be an effective, convenient, and supplementary method to differentiate thyroid nodules (10). Moreover, some studies had reported that qualitative color pattern SWE presents good repeatability and diagnostic performance in the diagnosis of breast masses (11, 12). To the best of our knowledge, the use of qualitative color pattern SWE to differentiate benign and malignant ACR TI-RADS 4 or 5 category thyroid nodules measuring ≤10 mm has rarely been reported.

The differentiation of ACR TI-RADS 4 or 5 category thyroid nodules measuring ≤10 mm is difficult, and quantitative and qualitative SWE could provide the stiffness information of thyroid nodules, which might be useful to differentiate benign and malignant thyroid nodules. This study aims to evaluate the diagnostic performance of quantitative SWE and a new qualitative color pattern SWE for the differentiation between benign and malignant ACR TI-RADS 4 or 5 category thyroid nodules measuring ≤10 mm and to find a non-invasive and useful method to improve diagnostic performance, which might be helpful for thyroid nodule management.

## 2 Materials and methods

This prospective study was approved by the ethics committee of Yueyang Central Hospital, and all patients signed informed consent before surgery.

### 2.1 Patients

**Figure 1:**
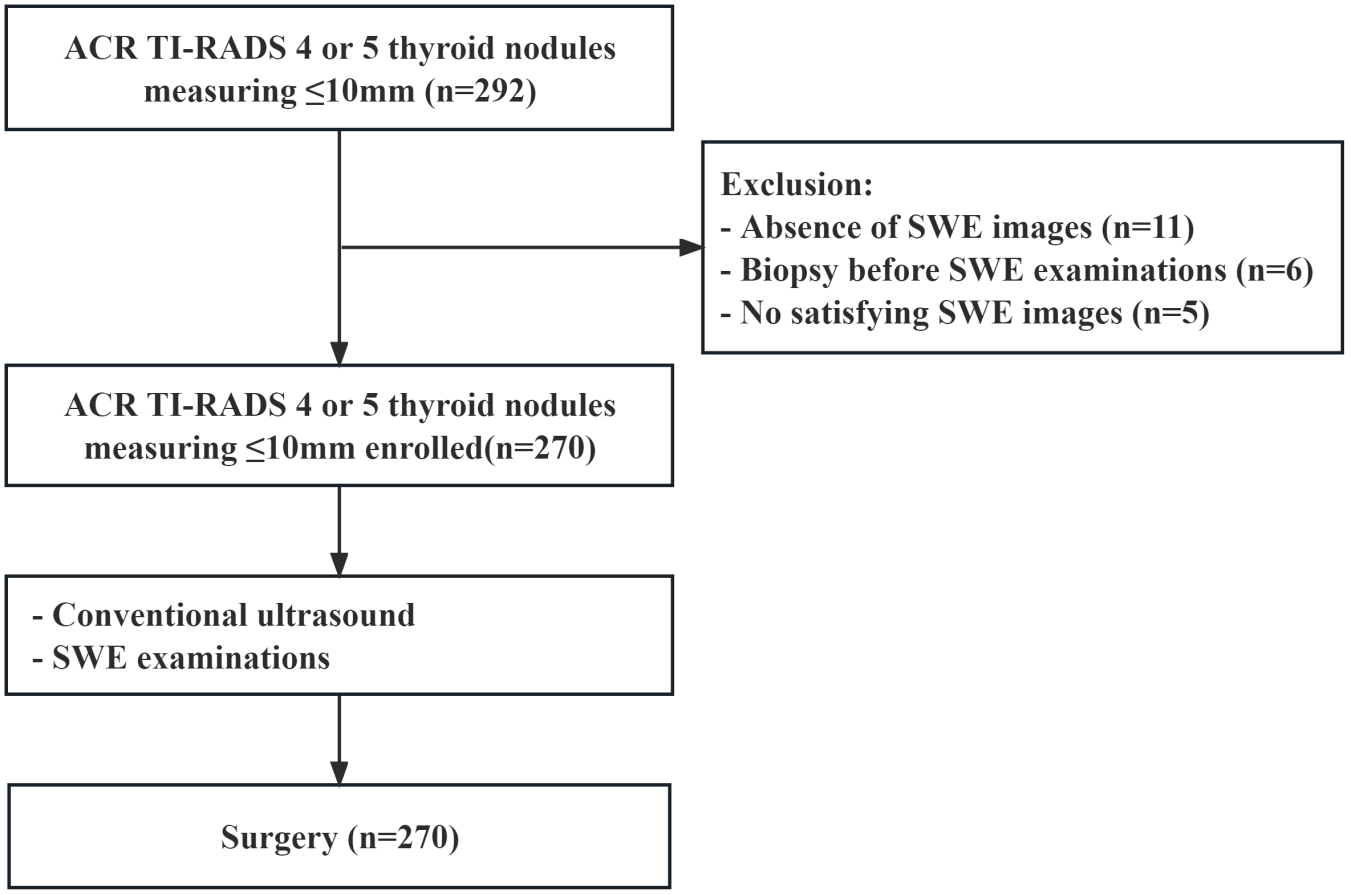
Flowchart of the patients enrolled.

From May 2020 to July 2022, a total of 237 patients with 270 thyroid nodules were enrolled (**Figure 1**). The inclusion criteria were as follows: (a) the pathology of all thyroid nodules were confirmed via surgery, (b) all patients underwent thyroid surgery within 1 month after SWE examination, (c) their age was 18 years or older, and (d) the greatest dimension of all thyroid nodules was ≤10 mm. The exclusion criteria were as follows: (a) patients had a previous biopsy, (b) patients had previous ablation, and (c) thyroid nodules without satisfied SWE images.

### 2.2 Research method

An Aixplorer ultrasound system (Supersonic Imaging, France) was used in this study. All thyroid nodules were screened by the same investigator with more than 10 years of experience in thyroid conventional ultrasound and 5 years of experience in SWE examination.

During conventional ultrasound, the patients were asked to maintain in the supine position with full exposure of the neck region. We used L15-4 or L10-5 linear array transducer to perform conventional ultrasound. The general characteristics of the thyroid nodules, such as location, nodule size, composition, echogenicity, shape, margin, and echogenic foci, were recorded in detail.

For the SWE examination, after conventional ultrasound examination, SWE was performed with L10-5 linear array transducer, while the patients were asked to hold their breath for several seconds until satisfied and stable SWE images were obtained. Quantification box (Q-box) was used for obtaining elasticity parameters, including *E*_max_, *E*_mean_, *E*_min_, *E*_ratio_, and standard deviation (SD). The diameter of Q-box was 2 mm. The first Q-box should be placed in the hardest region of the thyroid nodules, obtaining *E*_max_, *E*_mean_, *E*_min_, and standard deviation (SD). The second Q-box should be placed in the surrounding normal thyroid tissue, and *E*_ratio_ was obtained by calculating the ratio of the two Q-boxes. We have taken the median elasticity parameters of five measurements.

### 2.3 SWE image analysis

**Figure 2:**
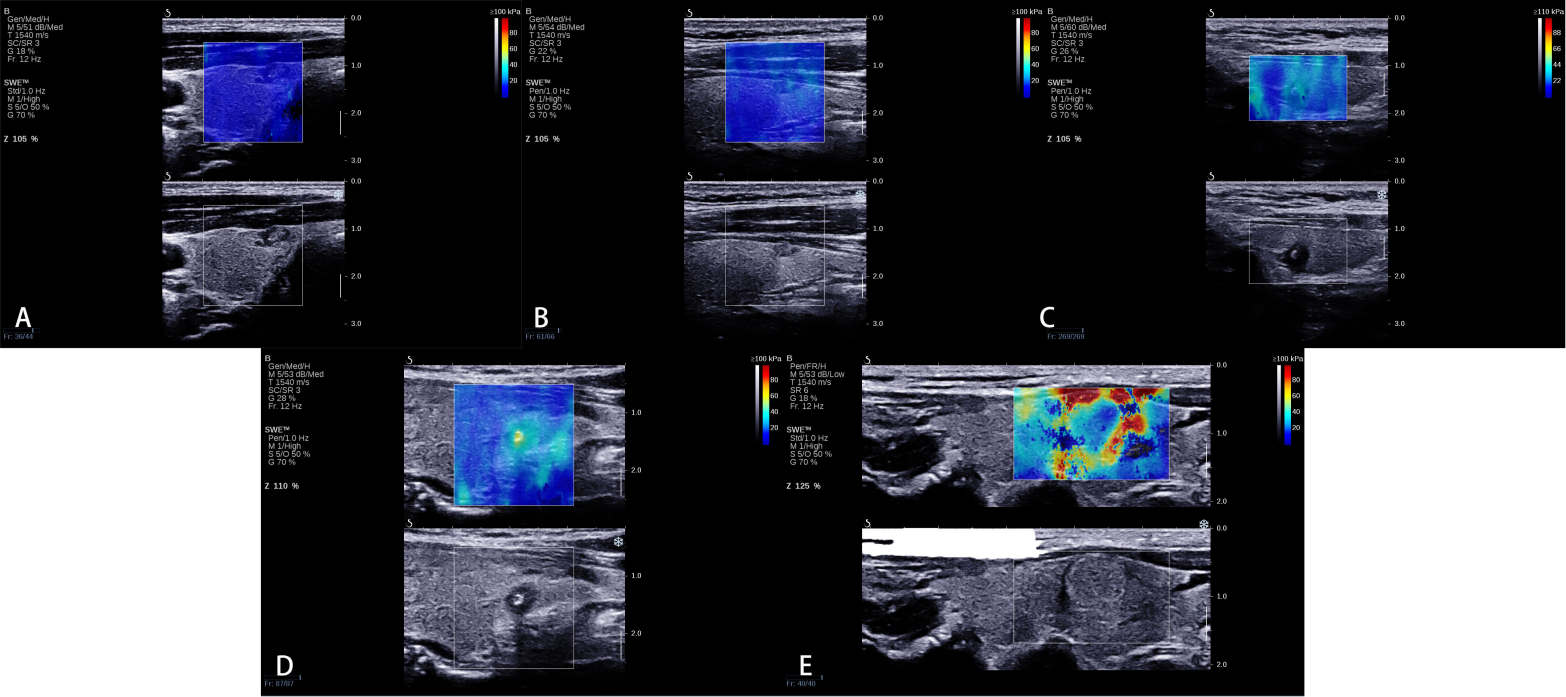
A new qualitative color pattern shear wave elastography (SWE) classification. **(A)** Pattern 1: the SWE color is homogeneously blue at the margin and inside the nodule, **(B)** pattern 2: the hardest color inside the nodule is green and the main color inside the nodule is blue, **(C)** pattern 3: the hardest color inside the nodule is green and the main color inside the nodule is also green, **(D)** pattern 4: the hardest color inside the nodule is yellow or red, and **(E)** pattern 5: a localized colored rim appears at the margin of the nodule, and the color of the rim is mainly yellow and red, which is called stiff rim sign.

We proposed a new qualitative color pattern SWE classification (**Figure 2**) in this study, which was classified into five patterns: (1) pattern 1: the SWE color is homogeneously blue at the margin and inside the nodule; (2) pattern 2: the hardest color inside the nodule is green and the main color inside the nodule is blue; (3) pattern 3: the hardest color inside the nodule is green and the main color inside the nodule is also green; (4) pattern 4: the hardest color inside the nodule is yellow or red; and (5) pattern 5: a localized colored rim appears at the margin of nodule, and the color of the rim is mainly yellow and red, which is called stiff rim sign.

### 24 Statistical analysis

SPSS 23.0 (SPSS, Chicago, IL, USA) and MedCalc 19.0 (MedCalc Software, Mariakerke, Belgium) were used to perform the statistical analyses. Univariate analysis was performed with independent *t*-test for continuous variables and chi-square test for categorical variables. Receiver operating characteristic (ROC) curves to differentiate benign and malignant thyroid nodules were drawn based on Young’s modulus of each thyroid nodule. The best cutoff value of Young’s modulus and area under curve (AUC) were calculated. The sensitivity, specificity, positive predictive value (PPV), negative predictive value (NPV), accuracy, and AUC of SWE and the combination of ACR TI-RADS and SWE were calculated and compared, respectively. A *P*-value <0.05 was considered to be statistically different.

## 3 Results

### 3.1 General information and pathological results

Among 270 nodules in 237 patients, 72 (26.67%) thyroid nodules were benign and 198 (73.33%) thyroid nodules were malignant. The general information and conventional ultrasound features are listed in **Table 1**. Age, shape, margin, echogenic foci, and ACR TI-RADS category were significantly different between benign and malignant thyroid nodules. Other general and conventional ultrasound features between benign and malignant thyroid nodules were not statistically different.

**Table 1:** General information, quantitative shear wave elastography (SWE) parameters, and qualitative SWE color patterns between benign and malignant thyroid nodules.

		Benign	Malignant	Value	*p*
Sex	Male	16	34	0.893	0.345
	Female	56	164		
Age	≤55	40	150	10.335	0.001
	>55	32	48		
Composition	Mixed cystic and solid	1	0	/	0.267
	Solid	71	198		
Echogenicity	Hyperechoic or isoechoic	2	0	/	0.070
	Hypoechoic or very hypoechoic	70	198		
Shape	Wider than tall	43	28	56.600	0.000
	Taller than wide	29	170		
Margin	Smooth	42	76	17.548	0.000
	Ill-defined	11	14		
	Lobulated or irregular	19	108		
Echogenic foci	None or large comet-tail artifacts	43	86	28.181	0.000
	Macrocalcifications	9	5		
	Peripheral calcifications	2	0		
	Punctate echogenic foci	18	107		
ACR TI-RADS category	4	32	42	14.324	0.000
	5	40	156		
Quantitative SWE parameters	*E*_min_	17.169 ± 8.313	23.025 ± 12.799		0.000
	*E*_mean_	23.979 ± 10.195	33.218 ± 17.054		0.000
	*E*_max_	32.424 ± 17.696	44.321 ± 24.154		0.000
	*E*_ratio_	1.376 ± 0.579	1.755 ± 0.813		0.000
	SD	3.201 ± 2.219	4.830 ± 3.653		0.000
Qualitative color pattern SWE	Pattern 1	12	14	54.705	0.000
	Pattern 2	30	15		
	Pattern 3	19	73		
	Pattern 4	9	84		
	Pattern 5	2	12		

### 3.2 Quantitative SWE parameters between benign and malignant ACR TI-RADS 4 or 5 category thyroid nodules measuring ≤10 mm

There were significant differences for all quantitative SWE parameters between benign and malignant ACR TI-RADS 4 or 5 category thyroid nodules measuring ≤10 mm. The best cutoff values for the differentiation of benign and malignant thyroid nodules were 18.25 kPa for *E*_min_, 28.15 kPa for *E*_mean_, 40.40 kPa for *E*_max_, 1.75 for *E*_ratio_, and 4.25 kPa for SD, respectively. The diagnostic performance of *E*_min_, *E*_mean_, *E*_max_, *E*_ratio_, and SD, respectively, is listed in **Table 2**. There was no significant difference among all quantitative SWE parameters.

**Table 2:** The diagnostic performance of quantitative shear wave elastography (SWE) parameters and qualitative color pattern SWE to differentiate benign and malignant American College of Radiology Thyroid Imaging, Reporting, and Data System 4 or 5 thyroid nodules measuring ≤10 mm.

Quantitative SWE parameters	Sensitivity (%)	Specificity (%)	PPV (%)	NPV (%)	Accuracy (%)
*E*_min_	62.63	63.89	82.67	38.33	62.69
*E*_mean_	55.05	72.22	84.50	36.88	59.63
*E*_max_	50.00	81.94	88.39	37.34	58.52
*E*_ratio_	41.92	86.11	89.25	35.03	53.70
SD	49.49	77.78	85.96	35.90	57.04
Qualitative color pattern SWE	85.35	58.33	84.92	59.15	78.15

### 3.3 Qualitative color pattern SWE between benign and malignant ACR TI-RADS 4 or 5 category thyroid nodules measuring ≤10 mm

There were significant differences in different qualitative color pattern SWE between benign and malignant ACR TI-RADS 4 or 5 category thyroid nodules measuring ≤1 cm. Benign thyroid nodules mainly present pattern 1 and 2, while malignant thyroid nodules always present patterns 3, 4, and 5. The sensitivity, specificity, PPV, NPV, accuracy, and AUC were 85.35%, 60.00%, 84.92%, 59.15%, 78.15%, and 0.740 (95% CI: 0.683–0.791), respectively. Compared with quantitative SWE parameters, qualitative color pattern SWE had better diagnostic performance with high sensitivity, which was significantly different.

### 3.4 Combination of qualitative color pattern SWE and ACR TI-RADS for the differentiation of ACR TI-RADS 4 or 5 category thyroid nodules measuring ≤10 mm

**Figure 3:**
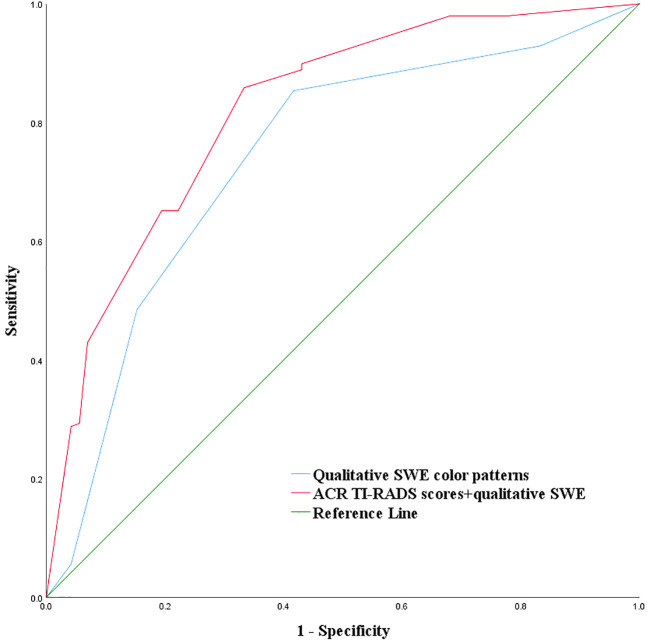
Combination of qualitative color pattern shear wave elastography and American College of Radiology Thyroid Imaging, Reporting, and Data System (ACR TI-RADS) for the differentiation of ACR TI-RADS 4 or 5 category thyroid nodules measuring ≤10 mm.

The criteria of combining qualitative color pattern SWE with ACR TI-RADS were as follows: Zero point was assigned for thyroid nodules presenting patterns 1 and 2 in qualitative color pattern SWE, while thyroid nodules presenting patterns 3, 4, and 5 were assigned three points. When assessing a thyroid nodule with combination of qualitative color pattern SWE and ACR TI-RADS, conventional ultrasound features and qualitative color pattern SWE were evaluated and summed up for the corresponding final point. With the optimal cutoff value of the total points ≥8, thyroid nodules were considered malignant. The sensitivity, specificity, accuracy, and AUC were 89.90%, 56.94%, 81.11%, and 0.820 (95% CI: 0.768–0.864), respectively (**Table 3**). Compared with using qualitative color pattern SWE alone, the combination of qualitative color pattern SWE and ACR TI-RADS had better diagnostic performance, which was significantly different (**Figure 3**).

**Table 3:** Combination of qualitative color pattern shear wave elastography (SWE) scores and American College of Radiology Thyroid Imaging, Reporting, and Data System (ACR TI-RADS) scores for the differentiation of ACR TI-RADS 4 or 5 category thyroid nodules measuring ≤10 mm.

Method	Criteria	Pathological results		Sensitivity	Specificity	Accuracy	AUC	*P* ^a^
		Benign	Malignant					
Qualitative color pattern SWE	Pattern 1 or 2	42	29	85.35	58.33	78.15	0.740 (0.683–0.791)	0.0249
	Pattern 3, 4, or 5	30	169					
Qualitative color pattern SWE scores + ACR TI-RADS scores	<8	41	20	89.90	56.94	81.11	0.820 (0.768–0.864)	–
	≥8	31	178					

a Comparison of the diagnostic performance of qualitative color pattern SWE with a combination of qualitative color pattern score SWE + ACR TI-RADS scores.

## 4 Discussion

In this study, we proposed a new qualitative color pattern SWE classification, which was useful to differentiate ACR TI-RADS 4 or 5 category thyroid nodules measuring ≤10 mm. Compared with quantitative SWE parameters, qualitative color pattern SWE had better diagnostic performance. Moreover, combining qualitative color pattern SWE with ACR TI-RADS could improve the sensitivity and accuracy compared with using qualitative SWE color patterns alone.

Currently, FNA is the most practical and useful method to get a definitive diagnosis before surgery. The diagnosis of FNA is based on the Bethesda System for Reporting Thyroid Cytopathology (TBSRTC). The third edition of TBSRTC in 2023 (13) is built on the success of the first two editions of TBSRTC in 2010 and 2017. The risk of malignancy (ROC) in the 2023 edition TBSRTC for Bethesda III, IV, and V category thyroid nodules are 13%–30%, 23%–34%, and 67%–83%, respectively. Thus, the diagnosis of Bethesda III and IV category thyroid nodules might be ambiguous. Moreover, small thyroid carcinoma ≤10 mm might mainly cause the “epidemic” of thyroid cancers (14), and thyroid nodules ≤10 mm were not routinely recommended for FNA according to ACR TI-RADS in 2017 (2), which was a challenge for the management of suspicious thyroid nodules ≤10 mm.

ACR TI-RADS 4 or 5 category thyroid nodules measuring ≤10 mm were difficult to distinguish. There were overlaps between benign and malignant thyroid nodules, which increased the difficulty in thyroid nodule diagnosis and management. Some studies found that the 2017 edition of ACR TI-RADS category had high diagnostic performance, and the ACR TI-RADS 5 category had higher sensitivity and lower specificity in the differentiation of benign and malignant thyroid nodules (15, 16). In this study, the overall malignant rate of thyroid nodules in ACR TI-RADS 4 and 5 category was 73.34%, while the sensitivity and specificity of ACR TI-RADS 5 category for distinguishing benign and malignant thyroid nodules were 78.79% and 44.44%, respectively, which was consistent with a previous study.

Quantitative SWE parameters had been proven useful in the differentiation of benign and malignant thyroid nodules (9, 17, 18). However, there were still some controversial problems, such as the lack of a consensus on optimal quantitative parameters and best cutoff values for the optimal quantitative parameters, the influence of nodule size on quantitative SWE parameters for the differentiation of benign and malignant thyroid nodules, and the discrepancy of quantitative measurement method in different studies. Previous studies had reported the value of quantitative SWE parameters in the differentiation of benign and malignant thyroid nodules measuring ≤10 mm. Gao et al. (18) reported that there was no statistical difference with *E*_max_ ≥51 kPa to differentiate thyroid nodules between thyroid nodules measuring ≤10 and >10 mm, and *E*_max_ had better diagnostic performance compared with *E*_mean_ and *E*_ratio_. Chambara et al. (19) found that no quantitative SWE parameter was significantly different between benign and malignant thyroid nodules. In this study, all quantitative SWE parameters were significantly different between benign and malignant ACR TI-RADS 4 or 5 category thyroid nodules; however, the diagnostic performance of all quantitative SWE parameters was unsatisfying. There was no statistical difference between all quantitative SWE parameters.

Compared with quantitative SWE parameters, qualitative elastography was less studied for the differentiation of benign and malignant thyroid nodules. Sengul et al. found that high SE scores might have high diagnostic performance in thyroid nodules with the cutoff over 15 mm compared with the cutoff over 10 mm (20). Moreover, several studies found that qualitative color pattern SWE was useful to distinguish benign and malignant thyroid nodules (10, 17). Tan et al. (17) found that the stiff rim sign was more likely to occur in malignant nodules. Zhang et al. (10) reported that qualitative SWE color scores could assist in differentiating thyroid nodules. In this study, we proposed a new qualitative color pattern SWE classification for the differentiation of benign and malignant ACR TI-RADS 4 or 5 category thyroid nodules measuring ≤10 mm. Using this classification, benign thyroid nodules mainly present patterns 1 and 2, while malignant thyroid nodules always present patterns 3, 4, and 5, which showed higher sensitivity and accuracy compared with quantitative SWE parameters.

There were 29/198 (14.65%) malignant thyroid nodules presenting patterns 1 and 2, and 51.72% (15/29) of these nodules measured ≤5 mm. The fiber components were relatively little in these small nodules, which caused less hardening. However, as the thyroid nodules grow larger, there is an increase of fibrous composition and calcification, and the nodules might be harder. Moreover, when ACR TI-RADS 4 or 5 category thyroid nodules measuring ≤1 cm present patterns 3, 4, and 5, it indicated that these thyroid nodules were hard, even if these thyroid nodules were small. Using this criterion, it had high sensitivity (85.35%) for predicting malignant nodules, which could be useful in clinical application based on high sensitivity.

The stiff rim sign of qualitative color pattern SWE was firstly proposed by Zhou et al. (21) for the differentiation of benign and malignant breast lesions, which indicated that the surrounding tissue of lesions had greater hardness, and it might be related to tumor infiltration and desmoplastic reaction into the surrounding tissue of lesions. Several studies used this sign in the differentiation of benign and malignant thyroid nodules (10, 17). Tan et al. (17) found that the stiff rim in thyroid nodules was more likely to occur in malignant thyroid nodules, and the malignant rate of thyroid nodules that present a stiff rim sign was 93.10%. Zhang et al. (10) reported that the malignant rate of thyroid nodules with a stiff rim sign was 92.9%, which also showed that the stiff rim sign was an important indicator for predicting malignant thyroid nodules. In this study, 85.71% (12/14) of the thyroid nodules with a stiff rim sign were malignant, which was consistent with previous studies. The reason why the malignant rate was slightly lower than previous studies might be because the thyroid nodules enrolled in this study measured ≤10 mm in size.

There are a few studies (22, 23) which reported the additional value of SWE on TI-RADS classification. Zhang et al. (22) found that the combination of *E*_max_ and ACR TI-RADS could improve diagnostic efficiency, especially for ACR TI-RADS 3 and 4 category thyroid nodules. Yang et al. (23) reported that the TI-RADS classification system modified by *E*_mean_ was more significant in the differentiation of benign and malignant thyroid nodules. In this study, we found that the qualitative color pattern SWE was significantly different between benign and malignant ACR TI-RADS 4 or 5 category thyroid nodules measuring ≤10 mm, which indicates that the qualitative color pattern SWE could reflect the stiffness information of thyroid nodules. Thus, we assigned the qualitative color pattern SWE point like the conventional ultrasound features in ACR TI-RADS: 0 point was assigned for pattern 1 or 2, and 3 points were assigned for pattern 3, 4, or 5. Adding together the qualitative color pattern SWE scores and ACR TI-RADS scores and using the optimal cutoff value of the total points ≥8, the combination of qualitative color pattern SWE and ACR TI-RADS had a higher AUC than using qualitative color pattern SWE alone for the differentiation of benign and malignant ACR TI-RADS 4 or 5 category thyroid nodules measuring ≤10 mm.

Moreover, the combination of qualitative color pattern SWE and ACR TI-RADS might be helpful for the management of thyroid nodules. FNA or further treatment might be suitable for patients with huge psychological pressure when the total point is ≥8 combining qualitative color pattern SWE and ACR TI-RADS.

There were several limitations in this study. First, this is a single-center study; a multi-center study with a large sample size should be conducted in the future. Second, all thyroid nodules were sequentially enrolled; however, only partial thyroid nodules were confirmed by surgery, which might cause inevitable sampling bias. Third, the time span of this study was short, and the pathological types of thyroid cancer were limited.

## 5 Conclusion

In conclusion, the combination of the new qualitative color pattern SWE and ACR TI-RADS had high sensitivity and accuracy, which might be a convenient and useful method to differentiate benign and malignant ACR TI-RADS 4 or 5 category thyroid nodules measuring ≤10 mm. It would be helpful for the management of thyroid nodules.

## Data availability statement

The raw data supporting the conclusions of this article will be made available by the authors, without undue reservation.

## Ethics statement

The studies involving humans were approved by the ethics committee of Yueyang Central Hospital. The studies were conducted in accordance with the local legislation and institutional requirements. The participants provided their written informed consent to participate in this study.

## Author contributions

AY: Formal analysis, Methodology, Writing – original draft, Writing – review & editing. WY: Data curation, Visualization, Writing – original draft. XC: Conceptualization, Data curation, Validation, Writing – review & editing. CD: Conceptualization, Formal analysis, Methodology, Writing – review & editing. BW: Conceptualization, Funding acquisition, Methodology, Supervision, Writing – review & editing.
